# SafetyCrown: a patient-centered, fully digital concept for immediate implant restoration following the one-abutment/one-time concept—a pilot case series of a new treatment concept

**DOI:** 10.1186/s40729-022-00434-2

**Published:** 2022-09-06

**Authors:** Lukas Waltenberger, Stefan Wolfart

**Affiliations:** Department of Prosthodontics and Biomaterials, Centre for Implantology, University Hospital Aachen, RWTH Aachen University, Pauwelsstr. 30, 52074 Aachen, Germany

## Abstract

**Introduction:**

The patient-centered SafetyCrown-workflow enables the immediate restoration of posterior missing teeth and short free-end situations following one-abutment/one-time within three visits and only one surgical approach. This prosthodontic rehabilitation aims to combine the advantages of cemented and screw-retained restorations.

**Report:**

The concept has been performed with 4 restorations in 3 patients and followed up for up to 1 year (mean: 11.2 months) without technical and/or biological complication. Visit 1: Intraoral optical impression, CBCT, and tooth shade selection. Virtual implant planning is performed, and a surgical guide is printed. After exporting the planned implant position, a tooth-colored abutment is fabricated from zirconia with a 1-mm supragingival cementation line, adhesively bonded to a titanium base. Visit 2: Fully navigated implant placement with insertion of the definitive abutment. Subsequently, optical impressions are prepared for A: immediate restoration using a PMMA crown without functional contacts; B: definitive crown fabricated from monolithic zirconia and individualized. The localization of the screw channel is marked using stain thus permitting precise screw channel access, if necessary. Visit 3: After osseointegration of the implant, the definitive crown is adhesively cemented supragingival. In a retrospective analysis of PROMs (‘How stressful was the treatment process […]?’ (0 = not stressful at all, 100 = very stressful), mean VAS score for SafetyCrown of 14 (SD 11.7) and 29.8 (SD 23.1) for standard procedure were present.

**Conclusion:**

The SafetyCrown offers a shortened, patient-oriented concept for implant-supported single-tooth reconstructions omitting second-stage surgery. Clinical performance and hypothesized prosthodontic benefits require confirmation via an RCT.

## Introduction

A missing posterior tooth is one of the most common clinical situations where a restoration with implant-supported single crowns is indicated. However, the standard treatment procedure from surgery to the insertion of the final restoration places high demands on the patients’ resilience, and the long-term stability of the peri-implant tissues is of great relevance to the procedure’s success.

There are several approaches for shortening the treatment time and/or improving patients’ perception and comfort. Under certain conditions, immediate restoration of the implant via a temporary restoration on a temporary abutment is possible. This shows similar long-term results as those associated with conventional loading for single crowns in the posterior area [1]. However, the key factor for the possibility of immediate restoration is sufficient primary stability using a high insertion torque (> 30 Ncm). Owing to developments in implant systems aiming for high primary stability, such as BoneLevelX (Straumann AG, Basel, Switzerland), Progressive Line (Camlog Biotechnologies AG, Basel, Switzerland), and Nobel Active and N1 (Nobel Biocare AG, Kloten, Switzerland), immediate restoration is more often possible and even predictable to a certain level.

In implant dentistry, the goal lies in the prevention of peri-implant diseases to ensure long-term success. Therefore, stable peri-implant soft tissue and the prevention of marginal bone loss are vital. The one-abutment/one-time concept was developed to preserve the soft and hard tissues. Per this concept, the definitive abutment is inserted either parallel to the implant placement or at the second stage of surgery after submerged healing. It prevents the use of healing abutments and the repeated replacement of abutments, each time accompanied by soft tissue trauma. This concept is promising and entails less marginal bone loss surrounding the implant [2–5]. However, few studies are available, and its clinical significance is uncertain. Targeting the one-abutment/one-time concept and reducing treatment duration, an innovative digital concept (Munic Implant Concept [6]) was developed and reported in 2015. An intraoperative digital impression is prepared after implant placement before submerged healing of the implant. Based on the digital impression, a definitive monolithic screw-retained crown is fabricated. It is inserted during second-stage surgery without the requirement of a healing abutment and multiple manipulations of the soft tissue. A retrospective patient study of the Munic Implant Concept with the use of a conventional impression posts documented a high level of patient satisfaction [7].

For the prosthodontic restoration of single crowns, a dentist can generally choose between screw-retained and cemented implant restorations. Several review articles have reported the survival rate and complication incidence of cemented and screw-retained implant reconstructions. Generally, the survival rates do not differ statistically between cemented and screw-retained reconstructions [8–10]. However, screw-retained reconstruction exhibits more technical challenges, with a 5-year complication rate of 24.4%, whereas cement-retained reconstruction accounts for 11.9%. Screw loosening is the most frequent technical challenge in single crowns. At 21.2%, it occurs significantly more often in screw-retained than in cement-retained crowns, where the complication rate is only 3.9%. Chipping of the veneering ceramic tends to occur more frequently in screw-retained crowns. Thus, the 5-year chipping rate is 9.6% for screw-retained and 2.8% for cement-retained suprastructures [8]. An analysis of the biological complications also revealed differences between cemented and screw-retained restorations. Marginal bone loss of more than 2 mm occurs more frequently during cementation (5-year incidence: 2.8%) than it does for screw-retained reconstructions (5-year incidence: 0%) [8]. Although high survival rates and low complication rates are reported, there is a need for further improvement in the restoration of single implant-supported crowns.

In this article, we present the SafetyCrown concept. It was developed to attain biological and prosthodontic long-term success for posterior single implants along with prosthodontic rehabilitation, as well as to increase patients’ perception of the treatment’s time efficiency. We aimed to achieve this by (1) limiting the surgical procedure; (2) shortening the treatment duration by immediate restoration; (3) aiming for minimal marginal bone loss and tissue perseverance with the one-abutment/one-time concept, and (4) implementing a hybrid crown design combining the advantages of screw-retained and cemented fixed implant reconstructions.

## Method

The method implementing the fully guided digital concept (SafetyCrown) is designed to offer patients in need of a single implant-supported restoration in the posterior area a safe and time-preserving treatment option, combined with the benefits of immediate restoration and the one-abutment/one-time concept.

**Fig. 1:**
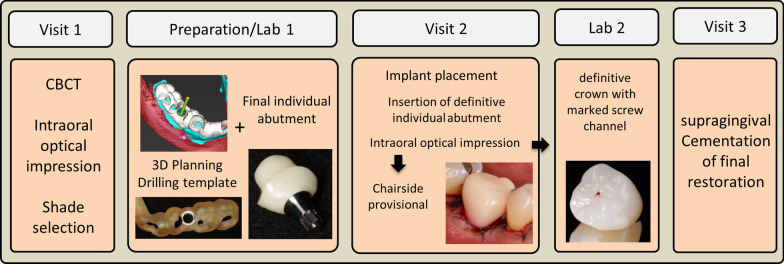
Visualization of the SafetyCrown-workflow, from the treatment planning to the insertion of the final crown

For the method itself, only three major visits are required. Figure 1 illustrates the workflow of the treatment method. It can also be implemented in the restoration of multiple implants, and tooth-retained fixed restorations. Complete diagnostics based on functional, periodontal, and full-mouth examinations as well as radiographic diagnostics are conducted in the planning phase. After patient information and shared decision-making, the SafetyCrown-workflow can commence.

### Visit 1

Full-arch intraoral optical impressions of the upper and lower jaws are prepared using an intraoral scanner. The individual shade of the adjacent teeth is selected and documented for an esthetically pleasing restoration. Furthermore, a cone-beam computer tomography (CBCT) is performed to assess the individual bone volume and for fully guided implant planning. The field-of-view (FOV) should be limited to the smallest FOV. Special focus should be placed on expectable artifacts limiting the possibility of precise matching.

### Preparation/laboratory

**Fig. 2:**
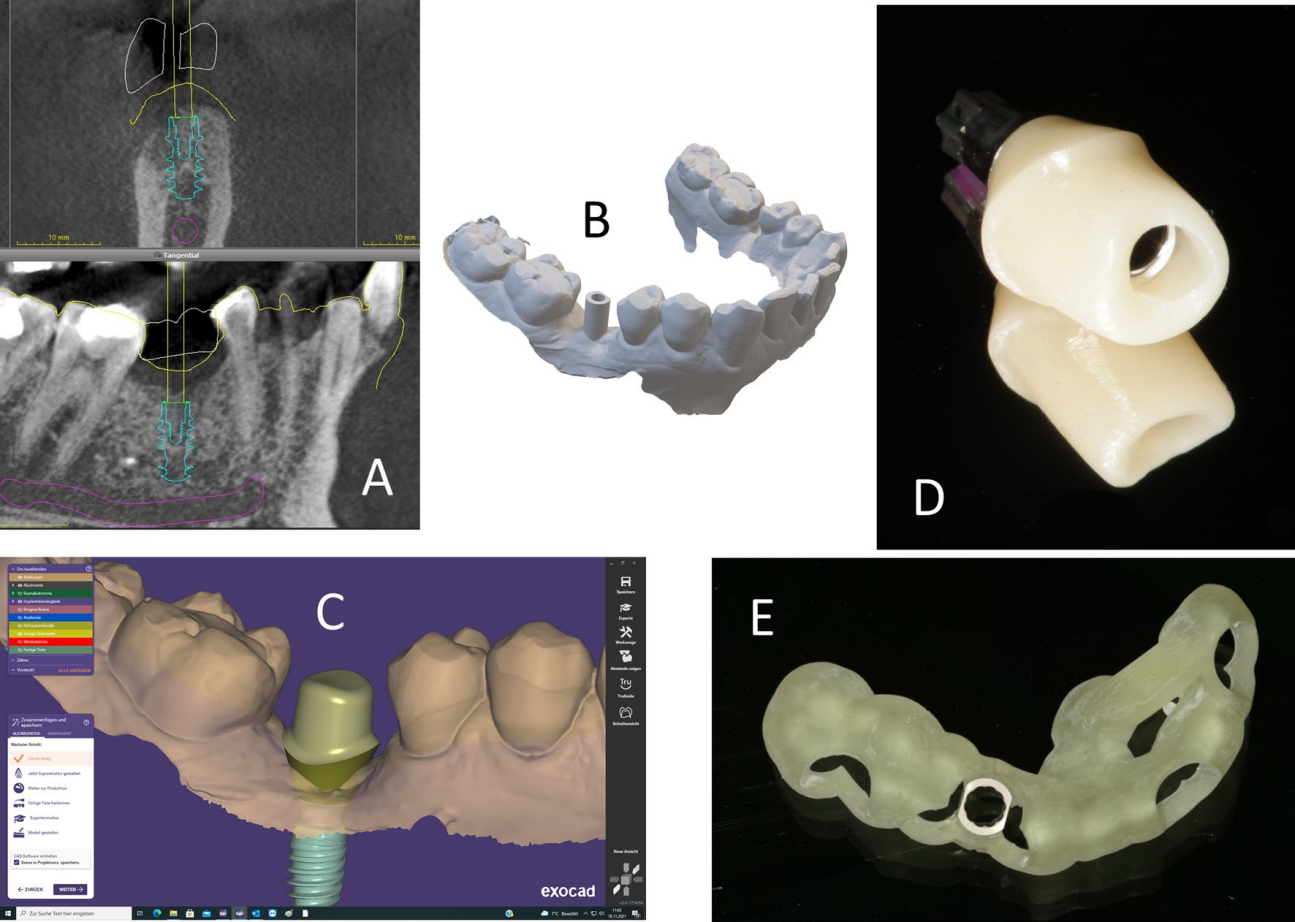
Preparation phase. **A** Planned implant aligned centrally in the axis of the tooth setup. **B** Virtual planning export. A scanbody represents the planned implant in position and index. **C** Design of the individual abutment with 1-mm supragingival cementation line. **D** Milled, polished and adhesively luted individual abutment on titanium base. **E** Fully navigated implant drilling template

Virtual models of the upper and lower jaws are generated based on the intraoral optical impressions and exported as standard tessellation language (stl) files. The CBCT data are exported as a Digital Imaging and Communications in Medicine (DICOM) dataset. For three-dimensional implant planning, a software with the possibility of exporting the planned implant position as an stl file can be used. The CoDiagnostiX software is used (Dental Wings, Montreal, Canada) by our working group. The DICOM-dataset is imported and matched with the virtual model. As a prosthodontic setup, a tooth is virtually placed in the edentulous area prior to the implant for ideal alignment of the screw channel (Fig. 2A). In functionally challenging occlusion patterns, an individual prosthodontic setup can be created in a laboratory computer-aided design/computer-aided manufacturing (CAD/CAM) software (Exocad 3.0, exocad GmbH, Darmstadt, Germany) and can be imported into the implant planning software. To fabricate the abutment, the implant position must be exported alongside the implant index. This can be represented via the placement of a scan body into the virtual model (Fig. 2B). In the next step, the exported model is imported into the laboratory-side CAD/CAM software (Exocad 3.0). An individual abutment is designed using tooth-colored, high-strength zirconia (LavaPlus, 3M, Saint Paul, Minnesota). To achieve a supragingival cementation line, the abutment is formed freely within the software, and the margin is not limited to the gingival height (Fig. 2C). After milling, sintering, and polishing according to the manufacturer’s specifications, it is adhesively bonded to a titanium base. Therefore, the titanium base as well as the zirconia abutment’s interface is sandblasted (Al_2_O_3_, 50 µm, 2 bar for titanium base, 1 bar for zirconia) and cleaned using an ultrasonic bath and 90% ethanol. Afterwards, a phosphate primer (Monobond Plus, Ivoclar Vivadent AG, Schaan, Liechtenstein) is applied to both surfaces and the zirconia abutment is adhesively bonded to the titanium base using a self-curing resin (Multilink-Hybrid-Abutment, Ivoclar Vivadent AG; Fig. 2D). In addition to manufacturing the abutment, a drilling guide is digitally designed within the implant planning software and fabricated using a three-dimensional (3D) printer or by milling. The drilling template must visualize the planned implant index position for correct orientation. Figure 2 illustrates the preparation steps as well as the individual abutment and implant drilling guide.

### Visit 2

**Fig. 3:**
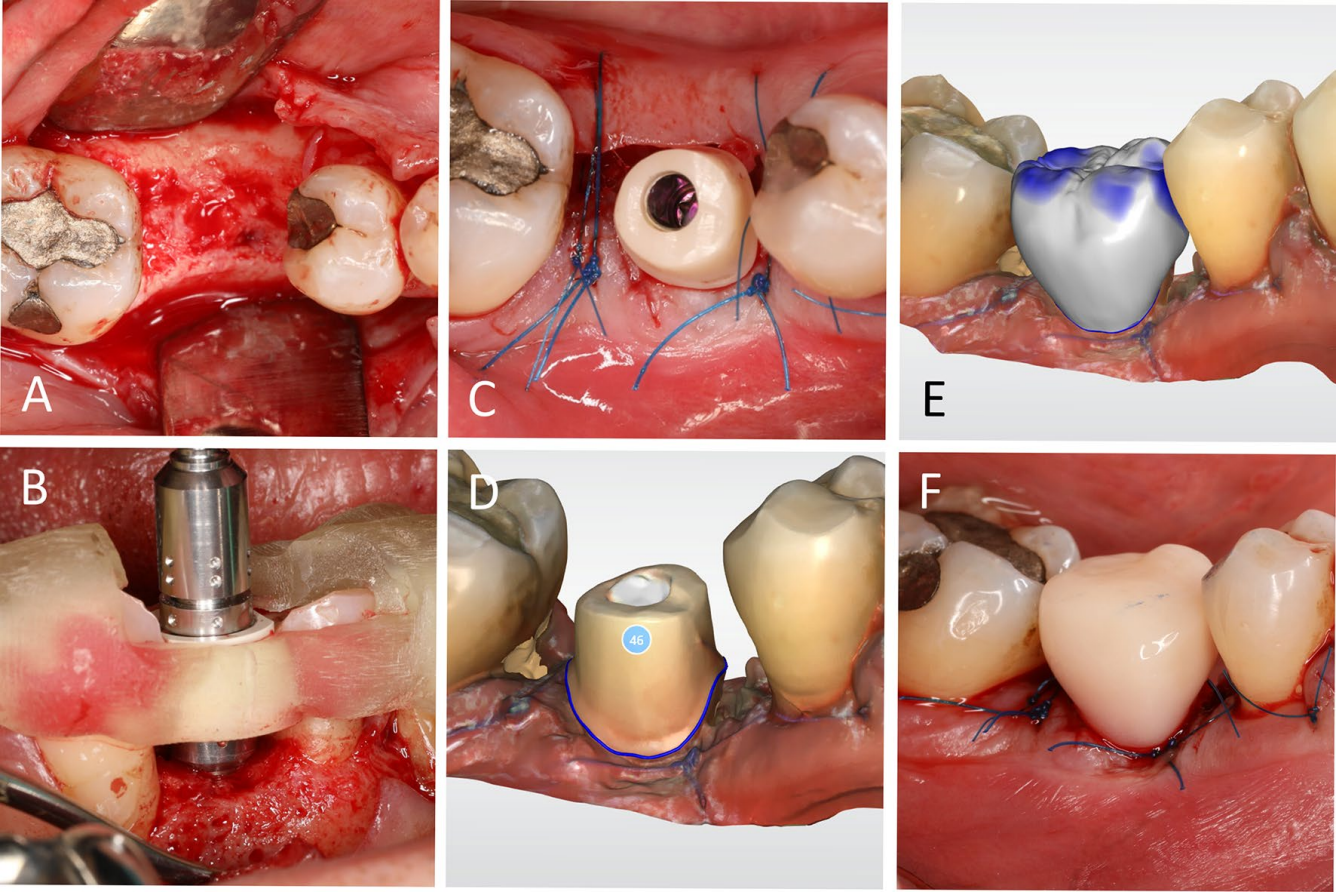
Implant placement and immediate restoration. **A** A full flap is elevated and the crestal bone is exposed. **B** Shows a properly aligned implant in terms of height and index. **C** The definitive abutment in situ and wound closure. **D** Intraoral optical impression in the region of the abutment. **E** Design of the temporary PMMA restoration. **F** Temporarily seated PMMA restoration

Implant placement is performed under local anesthesia. A full flap is elevated, and the crestal bone is exposed (Fig. 3A). Implant osteotomy is performed following the manufacturer’s guidelines, using a drilling guide aiming for sufficient primary stability of 35–50 Ncm. Figure 3B shows a properly aligned implant in terms of height and index. The abutment is tried in, and the screw is tightened. Small discrepancies in the vertical position, angle, and rotation are tolerable and can be compensated for by the temporary and final restorations. The soft tissue is adapted, and suturing is performed (Fig. 3C). The screw channel is provisionally closed using sterile Teflon tape. Afterwards, an intraoral optical impression of the abutment, opposing dentition, and buccal bite is taken (Fig. 3D). A chairside PMMA crown (Telio CAD, Ivoclar Vivadent AG) is designed (Fig. 3E) milled (MCXL, Dentsply Sirona, Bensheim, Germany) and provisionally cemented using Temp Bond NE (KerrHawe SA, Bioggio, Switzerland). Performing as an immediate restoration, it is assured that there are no occluding, dynamic, or approximal contact points (Fig. 3F).

### Laboratory

**Fig. 4:**
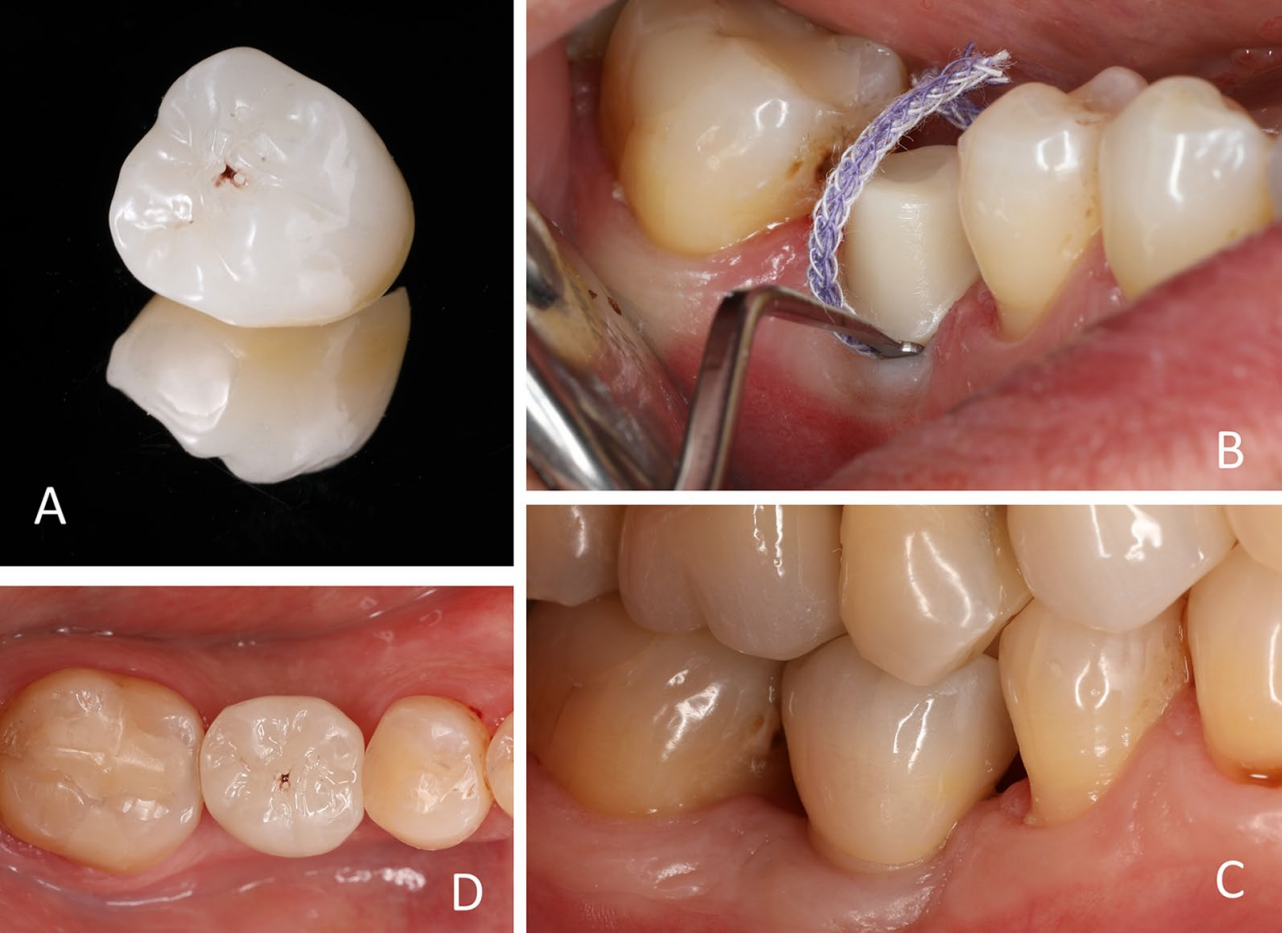
Definitive restoration. **A** The screw channel of the abutment is marked on the surface of the crown by stain, providing predictable access to the screw in case of complication. **B** For cementation, a thin retraction cord is placed just under the soft tissue line. **C**, **D** Seated definitive restauration

During implant healing, the definitive restoration with fully functional contacts is fabricated based on the digital impression of the abutment after insertion. It is milled out of tooth-colored, high-strength zirconia (IPS e.max ZirCAD Prime, Ivoclar Vivadent AG). As an important step, the position of the screw channel is marked by stain on the crown surface, providing predictable access to the screw in case of complications (Fig. 4A).

### Visit 3

**Fig. 5:**
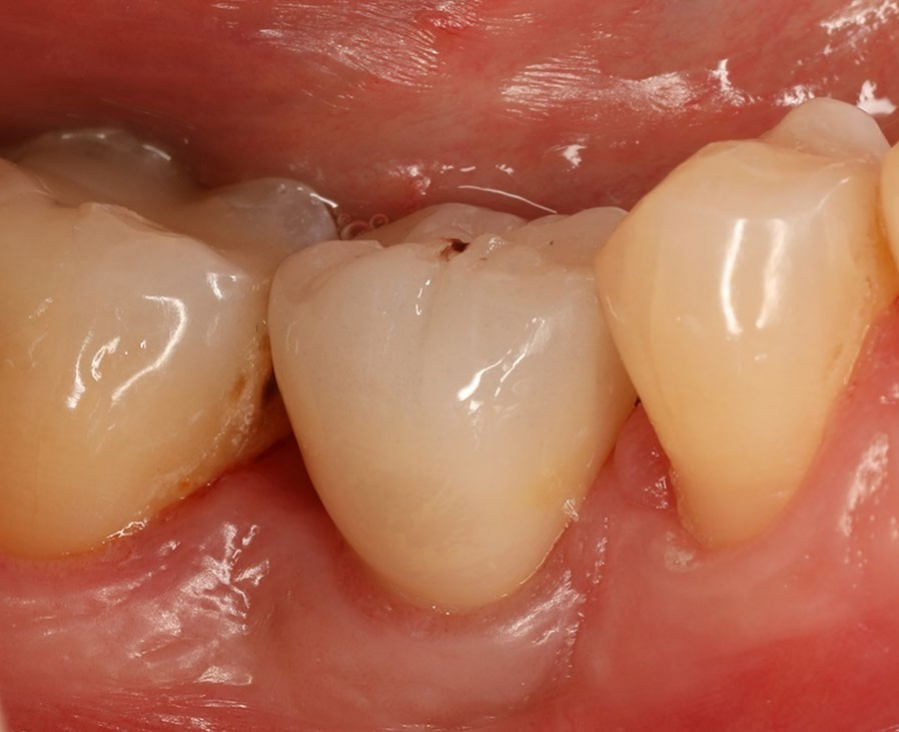
Follow-up 14 months after implant placement. A stable peri-implant soft tissue is present with no signs of inflammation

After permitting sufficient osseointegration of the implant, the provisional crown is removed, and the abutment is cleaned. The screw is retightened to the specified torque of 35 Ncm and the screw channel is sealed using sterile Teflon tape and a thin layer of bright composite resin. For cementation, a thin retraction cord is placed just under the soft tissue line (Fig. 4B). After extraoral sandblasting (1 bar, Al2O3 50 μm) and cleaning in an ultrasonic bath with 90% ethanol, the crown is adhesively cemented (Panavia 21 TC, Kuraray Noritake, Tokyo, Japan). The supragingival cementation line in combination with the thin retraction cord facilitates the removal of excess cement and a safe cementation process. Figure 4C, D illustrates the final restoration. Fourteen months after implant placement, a stable peri-implant tissue is present with no signs of inflammation (Fig. 5).

### Modification in case of multiple adjacent restorations

In case of several necessary adjacent restorations, it may be appropriate to fabricate the definitive crown part of the SafetyCrown together with the neighboring restorations. In these situations, the temporary restoration is removed after successful osseointegration, and the abutment is rescanned together with the adjacent preparations in an optical impression. The restorations can then be manufactured and seated simultaneously.

### Retrospective pilot study for sample size calculation

Based on this concept, three patients with 4 restorations were successfully treated (female, 53 years, FDI 47; female, 43 years, FDI 36; male, 53 years with two reconstructions, FDI 24, 46). They were in good general health, were non-smokers with no active periodontitis and did not suffer from bruxism. Implants were placed between 11 and 15 months ago and subsequently restored according to the protocol 3 months later (mean observation time 11.2 months). Until today no technical or biological complication occurred.

After the treatment, we enrolled a retrospective survey based on a questionnaire. To quantify the answers, we used a Visual Analog Scale (VAS) with scores ranging from 0 to 100 represented by a horizontal bar of 10 cm length. The individual answer was given by a vertical line. The questions were composed to gather more information about the patients’ perspective (Patient related Outcome Measures; PROMs) towards this new treatment concept. The questionnaire was completed by the three patients treated according to the SafetyCrown concept. As a control group, we randomly selected four patients from our clinic who recently had received an implant-supported posterior single crown using a conventional workflow (submerged healing, second-stage surgery, impression, try-in, insertion of the screw-retained crown). Based on this pilot study, we performed a sample size calculation to enroll an RCT with PROMs as primary outcome.

This retrospective survey was approved by the Institutional Review Board of the Medical Faculty, RWTH Aachen University (EK 069/22) and was conducted in accordance with the Helsinki Declaration of 1964, as revised in 2013. All patients provided written informed consent prior to completing the questionnaire.

The following question was asked in both groups:

1. How stressful was the treatment process from the implant placement to the definitive restoration with the implant-supported crown? (0 = not stressful at all, 100 = very stressful)

Only SafetyCrown:

2. How important did you consider the immediate restoration of the implant with a temporary during the waiting period to the definitive crown? (0 = totally unimportant, 100 = very important)
3. How much did the temporary improve your chewing ability? (0 = no improvement, 100 = great improvement)
4. How important did you consider the omission of the second surgical intervention? (0 = totally unimportant, 100 = very important)

Only control:

5. How stressful did you consider the remaining tooth gap after implant placement for the waiting period to the definitive crown? (0 = not stressful at all, 100 = very stressful)
6. How much did the remaining tooth gap influence your chewing ability? (0 = no negative influence, 100 = great influence)
7. How stressful did you consider the second surgical intervention? (0 = not stressful at all, 100 = very stressful)

## Results

The results of the VAS scores (range 0–100) with means and standard deviations are shown in Table 1. For question 1, mean VAS score of 14 (SD 11.7) for SafetyCrown and 29.8 (SD 23.1) for Control (Mann–Whitney *U*, *p* = *0.686*) were present.

**Table 1:** Results of VAS scores and standard deviations (SD) of questions 1 to 7 (range 0–100)

Question	SafetyCrown VAS score	Control VAS score
(1) How stressful was the treatment process from the implant placement to the definitive restoration with the implant-supported crown? (0 = not stressful at all, 100 = very stressful)	14 (SD 11.7)	29.8 (SD 23.1)
(2) How important did you consider the immediate restoration of the implant with a temporary during the waiting period to the definitive crown? (0 = totally unimportant, 100 = very important)	85.8 (SD 4.1)	
(3) How much did the temporary improve your chewing ability? (0 = no improvement, 100 = great improvement)	83.3 (SD 6.3)	
(4) How important did you consider the omission of the second surgical intervention? (0 = totally unimportant, 100 = very important)	70.5 (SD 22.5)	
(5) How stressful did you consider the remaining tooth gap after implant placement for the waiting period to the definitive crown? (0 = not stressful at all, 100 = very stressful)		36.3 (SD 35.1)
(6) How much did the remaining tooth gap influence your chewing ability? (0 = no negative influence, 100 = great influence)		55.3 (SD 30.2)
(7) How stressful did you consider the second surgical intervention? (0 = not stressful at all, 100 = very stressful)		47.8 (SD 39.0)

Questions 2–4 only SafetyCrown group; 5–7 only control group

### Sample size calculation

To calculate the sample size for an RCT investigating the SafetyCrown-Concept vs. a standard procedure with PROMs as primary outcome, G*Power 3.1.9.7 (HHU Düsseldorf, Germany) was used [11]. Based on the results (mean, SD) of the first question, a sample size of 19 patients per group (38 total) is necessary to achieve a power of 0.80 at a level of significance of 0.05 comparing both concepts.

## Discussion

The SafetyCrown concept was successfully implemented in a case study and thus revealed the following consequences for transferring the workflow to clinical practice: (1) it is possible to treat a patient with a posterior missing tooth and sufficient bone volume with only one operation in three visits via a fully digital workflow. It can be assumed that this reduction in treatment time, as well as the avoidance of additional surgical interventions, will lead to high patient-reported outcome measures (PROMs) [7]. (2) Maximum bone and peri-implant soft tissue preservation is achieved using the one-abutment one-time concept. (3) A high protection against chipping and screw loosening is achieved by the continuous unperforated occlusal surface of this supragingival cementation alternative. The risk of subgingival cement excess is minimized owing to the supragingival cementation line. (4) Due to the marking of the screw access, the perforation of the occlusal surface and thus weakening of the system only occurs in the event of a complication and not in general, as is the case with the occlusal screw system. This primarily considers the complication-free condition of the restoration, which has an estimated rate of 88.1% after 5 years, and not the more unlikely case of complication. Only in the more unlikely case of technical (11.8% in 5 years) or biological (2.8% in 5 years) complications, the SafetyCrown will have to be converted into a screw-retained restoration [8]. The clinical performance of the protocol as well as the prosthodontic benefits shown in this clinical case should be confirmed in an RCT.

The results of the retrospective survey indicate high importance of immediate restoration for the patients as well as a subjective improvement of chewing ability. The omission of second-stage surgery seems to be important, too. However, these results have to be interpreted with caution because of the retrospective design and the very low sample size. They were only collected to perform a sample size analysis, because no appropriate data about this topic could be found in the literature. According to the results of this pilot study, we plan an RCT with 20 patients for each group. There is a highly limited number of RCTs with PROMs as primary outcome [12] especially for single tooth reconstructions. Some of the recommendations of the EAO consensus statement [13] investigating PROMs of timing concepts in implant dentistry will be assessed in the upcoming RCT.

The implementation of the presented protocol is not universally applicable for missing single teeth as it involves limitations and requires certain conditions. Currently, the protocol is limited to a healed alveolar ridge without the need for major bone augmentation. If a hopeless tooth is present, bone healing should be awaited after extraction for predictable primary stability, followed by immediate restoration. Owing to the potential visibility of the supragingival cementation line, we currently limit this concept to the posterior area. The benefits and risks of an immediate restoration must be discussed with the patient. The high implant survival rate of 97.9% with a mean follow-up of 24.3 months is based on 1338 documented implants [1] and demonstrates that the immediate restoration of late-placed implants is a safe and predictable protocol.

Two potential concerns regarding SafetyCrown should be discussed. In cases where primary implant stability cannot be achieved, the protocol must be modified. We suggest that, after submerged healing, the abutment part of the SafetyCrown is inserted during the second-stage surgery. This procedure maintains the benefits of the one-abutment/one-time concept. After suturing, an intraoral optical impression is prepared, and the crown can be fabricated in analogy to the original protocol. As sufficient osseointegration is already present after submerged healing, the fabrication of a temporary restoration until near-term definitive restoration can be discussed with the patient. If the planned implant position has to be changed during the operation and fully guided implant placement is not possible, the abutment part of SafetyCrown can still be used in many cases. Special attention should be given to the index position with freehand implant placement. The protocol cannot be further followed in cases where a strong angular offset in the mesial and distal directions is present. Neighboring teeth could interfere with the insertion direction of the abutment.

We selected dental implants from the Straumann BLX-Series for this restoration method for two major reasons: (A) the progressive implant design enables predictable primary stability and therefore the possibility of immediate restoration, and (B) the sixfold index position decreases the correction effort and risk of losing the primary stability while adjusting the implant shoulder to the bone height and the index to the correct position. Generally, the SafetyCrown-workflow can be implemented with all dental implants approved for immediate restoration, offering guided surgery and the export of the planned implant position and index, as well as prosthodontic restoration with an individual abutment on a titanium base within a fully digital workflow.

The abutment design used in this method can be described as a hybrid abutment [14]. Monolithic high-strength zirconia was adhesively cemented to a titanium base. The implant–abutment connection is analogous to that observed in conventional prefabricated titanium abutments with similar mechanical properties [15]. Owing to the fabrication of the tooth-colored zirconia mesostructure inheriting similar translucency to that of dentin [16], it is superior to titanium in terms of esthetics, particularly in the presence of a recession around the abutment. For prosthodontic restoration, an individualized monolithic zirconia crown was then adhesively cemented on the hybrid abutment in this protocol. The safe supragingival cementation protocol inhibits subgingival cement residues. Two in vitro studies have indicated that the zirconia hybrid-abutment with a cemented crown might be superior in terms of failures compared to monolithic screw-retained zirconia crowns on titanium bases [17, 18]. As described in our protocol, screw access of the crown is maintained via surface markings on the crown. Based on this, we deduced superior mechanical properties for the prosthodontic restoration of the SafetyCrown-workflow with predictable screw access compared to that of cemented restorations, if necessary.

Comparing the treatment cost of the SafetyCrown method to a conventional submerged healed implant restored with a screw-retained single crown, the following can be summarized:

With the SafetyCrown treatment, costs can be decreased due to the reduced number of appointments (no second-stage surgery, no separate impression). Moreover, a healing cap and standardized gingival former are not necessary. Compared to a conventional crown, laboratory costs increase owing to the individual abutment and crown compared to a monolithic screw-retained restoration. The chairside temporary restoration additionally increases the expense of the SafetyCrown; however, it has a clear advantage for the patient. Considering this, we calculated a 10% increase in the overall treatment costs compared to that of a conventional restoration process.

## Conclusion

The presented SafetyCrown method enables a time-saving, patient-centered workflow for the restoration of implant-supported single crowns in the posterior region in three visits. It offers an immediate restoration, the benefits of the one-abutment/one-time concept, as well as a hybrid-abutment design combining the advantages of screw-retained and cemented restorations using an adhesively cemented crown with predictable screw access. However, its benefit in PROMs, clinical performance and long-term success require further evaluation in an RCT. The primary outcome of this RCTs will be PROMs and the sample size comprised 19 patients for each group (SafetyCrown concept versus standard concept) based on the performed pilot study.

## Abbreviations

CBCT: Cone beam computed tomography
CAD/CAM: Computer-aided design/computer-aided manufacturing
RCT: Randomized controlled trial
PROM: Patient-reported outcome measures
VAS: Visual Analog Scale
FDI: World Dental Federation
